# Accurate Diagnosis and Treatment of Painful Temporomandibular Disorders: A Literature Review Supplemented by Own Clinical Experience

**DOI:** 10.1155/2023/1002235

**Published:** 2023-01-31

**Authors:** Adam Andrzej Garstka, Lidia Kozowska, Konrad Kijak, Monika Brzózka, Helena Gronwald, Piotr Skomro, Danuta Lietz-Kijak

**Affiliations:** ^1^Department of Propaedeutic, Physical Diagnostics and Dental Physiotherapy, Faculty of Medicine and Dentistry, Pomeranian Medical University, Szczecin, Poland; ^2^Student Scientific Society, Pomeranian Medical University, Szczecin, Poland; ^3^Student Scientific Society, Medical University of Silesia, Zabrze, Poland

## Abstract

**Introduction:**

Temporomandibular disorders (TMD) is a multifactorial group of musculoskeletal disorders often with combined etiologies that demand different treatment plans. While pain is the most common reason why patients decide to seek help, TMD is not always painful. Pain is often described by patients as a headache, prompting patients to seek the help of neurologists, surgeons, and ultimately dentists. Due to the unique characteristics of this anatomical area, appropriate diagnostic tools are needed, as well as therapeutic regimens to alleviate and/or eliminate the pain experienced by patients. *Aim of the Study*. The aim of this study is to collect and organize information on the diagnosis and treatment of pain in TMD, through a review of the literature supplemented by our own clinical experience. *Material and Methods*. The study was conducted by searching scientific databases PubMed, Scopus, and Google Scholar for documents published from 2002–2022. The following keywords were used to build the full list of references: TMD, pain, temporomandibular joint (TMJ), TMJ disorders, occlusal splint, relaxing splints, physiotherapy TMD, pharmacology TMD, natural therapy TMD, diagnostic criteria for TMD, and DC/TMD. The literature review included 168 selected manuscripts, the content of which was important for pain diagnosis and clinical treatment of TMD.

**Results:**

An accurate diagnosis of TMD is the foundation of appropriate treatment. The most commonly described treatments include physiotherapy, occlusal splints therapy, and pharmacological treatment tailored to the type of TMD.

**Conclusions:**

Based on the literature review and their own experience, the authors concluded that there is no single ideal form of pain therapy for TMD. Treatment of TMD should be based on a thorough diagnostic process, including the DC/TMD examination protocol, psychological evaluation, and cone beam computer tomography (CBCT) imaging. Following the diagnostic process, once a diagnosis is established, a treatment plan can be constructed to address the patient's complaints.

## 1. Introduction

Temporomandibular disorders (TMD) can present with pain, prompting patients to seek help from various specialists [1–4]. TMD is most frequently seen in people aged between 20 and 40 years [5–8] and is more common in women due to hormonal changes and greater influence of psychosocial factors [9–12]. Thus, it can be concluded that TMD is a civilization problem, which may escalate due to the increasing pace of life, omnipresent stress, and improper use of the masticatory system [13–24]. One unquestionable causative factor is stress, which has a destructive effect on all masticatory structures, and if chronic, it may expose or aggravate temporomandibular disorders [25–32].

Pain in temporomandibular disorders may have a diverse etiology, i.e., central or peripheral, as demonstrated by the 2020 study by Yin et al., finding that TMD is accompanied by functional and structural changes in the primary somatosensory cortex, prefrontal cortex, and basal ganglia of the brain, which should inform treatment decisions [33]. Temporomandibular disorders (TMD) are characterized by abnormalities in the temporomandibular joint, masticatory muscles, and other adjacent structures, often described by patients as a headache [34–40]. According to research findings, typical TMD symptoms are more common in patients with migraine or tension headaches. It has also been shown that patients with diagnosed TMD are more likely to experience migraines, and the coexistence of both problems exacerbates the symptoms of each [41–47]. This unique anatomical region does not lend itself easily to diagnosis and treatment. It is not uncommon for patients to be referred to neurologists, otolaryngologists, surgeons, and dentists. Undoubtedly, the involvement of many specialists in the problems affecting this area may be beneficial in the classification and differentiation of disorders [48–51].

Masticatory dysfunction can be diagnosed when at least three of the following symptoms are reported: pain and acoustic symptoms during mandibular movements, limited mandibular mobility, difficulty with jaw opening, and occlusal or nonocclusal parafunction. The modern diagnosis of TMD should be based on the DC/TMD examination protocol because only with the correct diagnosis is the correct treatment possible [52–54].

## 2. Aim of the Study

The aim of this study is to collect and organize information on the accurate diagnosis and treatment of pain in TMD through a review of the literature supplemented by our own clinical experience.

## 3. Materials and Methods

The study was conducted by searching scientific databases PubMed, Scopus, and Google Scholar for documents published from 2002–2022. The literature review included 168 selected manuscripts, the content of which was important for pain diagnosis and clinical treatment of TMD. These aspects mentioned previously were the criteria for the inclusion of the manuscripts in the review. The following keywords were used to build the full list of references: TMD, pain, TMJ disorders, occlusal splints, relaxing splints, physiotherapy TMD, pharmacology TMD, and natural therapy TMD.

## 4. The Essence of the Matter

### 4.1. TMD Pain Diagnosis

#### 4.1.1. Myalgia

Myalgia (muscle pain) can be caused by mandibular movements, parafunctions, and excessive muscle tension due to the increased activity of masticatory muscles. Pain occurs upon provocation testing. The patient's history may include pain in the jaw, temple, ear, or in front of the ear. Pain may be modified with jaw movement, function, or parafunction.

Upon physical examination of the patient, the physician is able to confirm the location of pain in the temporalis or masseter muscle, additionally using muscle palpation and maximum unassisted or assisted jaw opening [55–75].

#### 4.1.2. Myofascial Pain

Myofascial pain can be local or referred to and is experienced by the patient as deep and dull. Unlike myalgia, this pain spreads beyond the palpated area, remaining inside the boundary of the examined muscle or in the case of referred myofascial pain–beyond the area of the examined muscle. Myofascial trigger points may also be felt during palpation [76–80].

#### 4.1.3. Arthralgia

The term arthralgia refers to pain in the temporomandibular joint without signs of joint inflammation. The onset of pain is associated with mandibular movement, function, and parafunction. Pain is also triggered during provocation testing. The patient's history includes pain in the jaw, temple, ear, or in front of the ear. On physical examination, the physician confirms pain in the TMJ area, especially the lateral region, and examines the maximum range of jaw opening with and without assistance [81–83].

#### 4.1.4. TMD-Attributed Headache

Headache attributed to temporomandibular dysfunction is characterized by a history of temporal pain of any nature. The pain can be modified by mandibular movement, function, and parafunction. Upon physical examination, pain in the temporalis region can also be observed in provocative tests. Pain may occur during palpation and when testing jaw opening [84].

#### 4.1.5. Disc Displacement with Reduction or with Intermittent Locking

An intracapsular disorder involving the condyle-disc complex. To make the diagnosis, it is necessary to determine the closed mouth position according to the protocol. At the Department of Propaedeutics, Physical Diagnostics, and Dental Physiotherapy, we ask the patient to assume their habitual occlusion and then relax the mandible. In this way, we are able to assess the actual intraarticular status, which is confirmed by palpation, joint sound inspection (with a stethoscope), and diagnostic imaging (CBCT). When performing diagnostic imaging, it is essential to perform the examination under the same conditions, without the bite stick.

In this disorder, the disc is positioned anteriorly relative to the condylar head and reduces with mouth opening movements. In some cases, medial and lateral displacement of the articular disc can be observed, as well as noises such as clicking, crackling, or popping [85–93]. Please note that if the patient has a history of joint locking and chewing problems, this diagnosis is ruled out.

To make the diagnosis, the patient is asked to report all TMJ noises that have occurred in the last 30 days during mandibular movements, and additionally, the patient should report any noises during the examination:

- Clicking, popping, and/or snapping noise during both opening and closing movements, detected with palpation during at least one of three repetitions of jaw opening and closing movements; or
- Clicking, popping, and/or snapping noise detected with palpation during at least one of three repetitions of opening or closing movement(s);
- Clicking, popping, and/or snapping noise detected with palpation during at least one of three repetitions of right or left lateral, or protrusive movement(s) [94–100].

When discussing this disorder, it should be stated that imaging should be the reference standard for this diagnosis [101–103].

#### 4.1.6. Disc Displacement without Reduction with Limited Opening

In this intracapsular disorder, in the closed mouth position, the disc is positioned anteriorly relative to the condylar head and does not reduce in size with the opening of the mouth. Characteristically, the disorder is associated with persistent limited mandibular opening, sometimes referred to as a closed lock, which is not resolved by a manipulative manoeuvre performed by the physician.

Patient history includes a locked jaw, limited movement, and eating difficulties. In physical examination, during assisted jaw opening, the distance between the upper and lower incisors is less than 40 mm. Passive movements may be accompanied by noise [104–107].

#### 4.1.7. Osteoarthritis of the Temporomandibular Joint

This disorder involves joint tissue deterioration with concomitant osseous changes in the condylar head and/or articular eminence.

In history, the patient reports noise when chewing or opening the mouth in the last 30 days, and these phenomena may also appear during the examination. On physical examination, the physician detects snapping, popping sounds in the joint during the abduction, adduction, and lateral or protrusive movements. Imaging is required, as CBCT may help visualize subchondral cysts, erosions, generalized sclerosis/calcification, or osteophytes [108–111]..

#### 4.1.8. Subluxation

A hypermobility disorder involving the disc-condyle complex and the articular eminence. In the open mouth position, the disc is anterior to the articular eminence and the normally closed mouth position cannot be restored without a manipulative manoeuvre. The difference between subluxation and luxation is that in the former the patient is able to reduce the dislocation on their own, whereas the latter requires professional intervention. Patient history includes jaw locking upon abduction movement in the last 30 days. These locks may have been incidental and temporary, resulting in an inability to close the mouth [112, 113].

The RDC/TMD and DC/TMD protocols make it possible to establish a diagnosis but do not shed any light on the etiology of the disorder, and elimination of the cause or an attempt to create the optimal conditions will be crucial in the treatment process.

At the Department of Propaedeutics, Physical diagnostics, and Dental Physiotherapy, the treatment team consists of an orthodontist, a physician dealing with dental prosthetics and restorative dentistry, a physiotherapist, and a dentist who coordinates the work of the whole team [114]. One of the most common signs of a disease process within the TMJ are sounds emitted by the articular structures, such as popping, clicking, humming, grinding, or crunching [114].

Egermark et al., after examining 320 children aged 7, 11, and 15 years, reported that acoustic symptoms were more common in those with malocclusion (24%), with a predominance of transverse malocclusion. In their conclusions, they noted that there were no significant differences in the prevalence of masticatory dysfunction in the studied population between patients with malocclusion and those with a normal bite [115].

Research findings provide no clear-cut conclusion as to how temporomandibular joint disorders are affected by a malocclusion. The consequences of malocclusion in terms of TMD development may be manifold and are undoubtedly related to age, gender, as well as the severity of the disorder.

A fairly significant problem reported and observed in patients is nocturnal bruxism, which affects 8% of the population, and awake bruxism, the prevalence of which is estimated at 20%. At present, bruxism is defined not as a disorder but as a physiological stress-coping mechanism [116–121].

Based on our own experience, we would like to note the relatively frequent coexistence of TMD with orthodontic disorders and temporomandibular disorders in post-orthodontic patients, where the teeth were often aligned in arches while the condylar heads were displaced posteriorly with reduced joint space [122]. In addition, it is important to consider that dental arches are somatic sites where excessive emotional tension can be diffused and reduced [123].

Research into the associations between malocclusion and TMD, as well as the influence of malocclusion treatments on TMD should be conducted in large study samples..

### 4.2. TMD Pain Therapy

#### 4.2.1. Natural Methods

Acupuncture is the best-known method of traditional Chinese medicine that is often used, also in Poland, in the treatment of chronic pain. Acupuncture points often coincide with so-called trigger points and correspond to sites of increased density of *A*-*δ* and *C* fibre nerve endings that conduct pain sensations. Warm compress therapy is used for chronic inflammation and muscle strains. Ideally, a warm compress at 35–40 degrees *C* should be applied for 20–30 minutes. Cold compresses, on the other hand, are good for acute inflammation with pain and swelling [124, 125].

#### 4.2.2. Psychological and Behavioural Methods

Psychological and behavioural programmes are effective in alleviating the psychological crisis, allowing the patient to change their perception of pain and improving functioning in patients with chronic pain. The therapeutic effect is not affected by the duration of the programme or by whether the treatment is delivered in an individual or group setting.

Behavioural approaches aim to reduce the frequency of pain-promoting behaviours and increase the frequency of health-promoting behaviours. They include:

- improving physical fitness
- social and employment activation
- reducing the amount of medication
- reducing overuse of health services

Psychological methods include the following:

- modifying ways of thinking about pain (misconceptions about pain) that cause prolonged suffering and disability
- replacing a sense of helplessness with a sense of control over pain and one's own life
- developing strategies for adequate and effective pain management
- returning to work and promoting an active lifestyle [126, 127]

It must be remembered that effective pain control requires a multidimensional approach, aiming to reduce the pain but also to improve the patient's quality of life.

#### 4.2.3. Interventional Methods-Splint Therapy

Occlusal splint therapy can be used in all TMD disorders; however, it is vitally important to use the right splint for the patient's unique situation.

An occlusal splint is an appliance that affects the mutual relationship of the upper and lower teeth and, consequently, the relationship of the condylar process to the mandibular fossa and articular eminence within the TMJ. The purpose of splints is to stabilize occlusion or to protect teeth from excessive abrasion [128, 129].

According to numerous studies, the use of splints has a significant effect on alleviating or even eliminating the patient's pain symptoms. In cases of disc displacement, repositioning splints are used to stabilize the mandible in the centric relation, and in cases of masticatory muscle disorders, relaxation splints are used to prevent parafunctional effects [130, 131].

Splints are most commonly made by obtaining dental impressions and making a bite registration with wax or silicone mass. An intraoral scanner and electronic bite registration can also be used.

The technique recommended by our team for making occlusal splints is 3D printing using special resin, which makes it possible to avoid the mistakes common in the conventional hand-made process. On the basis of our own experience, research findings, and patient feedback, we use two types of splints in the Department of Propaedeutics, Physical diagnostics, and Dental Physiotherapy: the Michigan-type relaxation splint and the maxillomandibular repositioning splint [132, 133].

The Michigan-type relaxation splint with canine guidance is used in cases involving: myalgia, myofascial pain, and TMD-attributed headache.

The relaxation splint is made from hard resin and always applied to a single arch, with the upper usually being the arch of choice–unless there are missing teeth in the back. Importantly, in the case of missing teeth, the design of the splint should allow for retention elements.

The hard repositioning splint joined interocclusal in the correct construction bite relationship is used in the following situations: arthralgia, disc displacement with reduction, disc displacement with reduction with intermittent locking, disc displacement without reduction with a limited opening, disc displacement without reduction and without limited opening, osteoarthritis of the temporomandibular joint, subluxation.

#### 4.2.4. Physiotherapy

Physiotherapy is a discipline of health science that aims to eliminate, alleviate, and prevent various ailments, as well as restore functional ability through movement and various physical agents. Physiotherapists are part of the treatment process in the case of dysfunctions involving the neuromuscular, musculoskeletal, and other systems [134].

In their work, physiotherapists use kinesiotherapy and physical therapy techniques.

- Self-therapy and muscle training. The patient is taught how to perform the correct opening, closing, lateral and protrusive movements of the mandible, as well as how to deal with sudden pain. Exercises should be performed daily in front of a mirror, and if the treatment includes a splint, it should also be used during exercises. The purpose of the exercises is to shorten the overstretched muscles and relax them, which may help improve symmetry and regulate muscle tone [135].
- Manual therapy makes use of trigger points. For disc displacement, a joint mobilization technique is applied, which involves the physiotherapist performing traction and gliding movements with low velocity but increasing amplitude. These movements are performed parallel and perpendicular to the joint surface. If the mandibular range of motion is limited, muscle energy techniques (MET) can be used. Treatments using the MET involve the repetition of three steps: in step one, the muscle is stretched to the point of resistance of the tissues; in step two, the patient slightly contracts muscles for about 10 seconds trying to resist the force generated by the physiotherapist; in the last step, the patient relaxes the muscles [136].
- Massage is used for myofascial pain in order to achieve pain relief and improve muscle length and flexibility, as well as loosen fascia [137, 138]. The frequency of massage sessions should be 30 minutes twice a week. With subsequent visits, the treatment should be applied with increasing force.
- Physical therapy, such as ultrasound and transcutaneous electrical nerve stimulation (TENS) can be used for pain of muscular origin. Therapeutic ultrasound can be applied in three modalities: using continuous waves, short bursts (pulsed ultrasound), and ultrasound combined with electrical stimulation, the latter of which has proven to be the most effective.

TENS relieves pain and relaxes masticatory muscles in symptomatic patients with TMD [139–142]. In the pain of intracapsular origin, positive results have been observed after the application of a magnetic field combined with LED light therapy. The Solux infrared lamp can be used in cases of arthropathy and rheumatic diseases. The beneficial effects of heat therapy include the alleviation of pain.

- The Kinesio Taping method is used for TMJ stabilization. It should be applied bilaterally. The tapes work by reducing the tension in the masticatory muscles, as well as the adjacent structures such as the muscles of the neck, shoulders, and spine [143–146]. The application of tapes also stimulates lymphatic drainage, which has a beneficial effect on inflammation accompanied by tissue swelling.
- Iontophoresis is the use of direct electrical current to accelerate the transdermal delivery of nonsteroidal anti-inflammatory drugs (NSAIDs), corticosteroids, and analgesics. While it is not associated with pain relief, a significant improvement in the range of motion in the joint has been observed [147].

#### 4.2.5. Pharmacotherapy

The decision on the use of medications in temporomandibular disorders should be preceded by a thorough analysis of the risks and benefits of the drug [148–152]. Medications used to treat TMD include analgesics, nonsteroidal anti-inflammatory drugs, anticonvulsants, muscle relaxants, and benzodiazepines [153, 154].

#### 4.2.6. Nonsteroidal Anti-Inflammatory Drugs (NSAID)

NSAIDs are beneficial for patients with acute temporomandibular arthritis resulting from sudden disc displacement. Treatment should continue for a minimum of two weeks, and it is important to combine NSAIDs with gastroprotective agents. Among NSAIDs, ibuprofen appears to be the safest for the gastrointestinal tract [155].

It should also be noted that taking NSAIDs for more than 5 days may reduce the efficacy of antihypertensive drugs, such as diuretics, beta-blockers, and ACE inhibitors [154, 155]. In addition, NSAIDs used with anticoagulants such as warfarin or acenocoumarol may increase the risk of bleeding.

#### 4.2.7. Opioids

Due to the interactions of NSAIDs with anticoagulants, as well as the risk of gastritis, physicians sometimes choose to administer oral opioids, such as codeine and oxycodone. The intraarticular delivery route has been studied, but the findings are conflicting [156]. It is essential to bear in mind the side effects of opioid use, which include: dizziness, excessive sedation, nausea, vomiting, constipation, physical dependence and addiction, and respiratory depression. Because of the mentioned reasons, the use of opioids for the management of TMD should be discouraged [157–159].

#### 4.2.8. Corticosteroids

Corticosteroids are helpful in the treatment of moderate to severe TMD. They can be administered by intraarticular injection or by oral route. They have an anti-inflammatory effect which can help relieve pain.

For intraarticular injections, it is a good idea to combine corticosteroid preparation with a local anaesthetic, such as lidocaine. According to research findings, this approach provides for a significant reduction in pain, lasting 4 to 6 weeks, and a reduced risk of complications.

Corticosteroids should be used with caution or discontinued in patients with hypertension, adrenal disease, or electrolyte problems. On day 4 after injection, it is recommended to introduce NSAIDs [160–163].

#### 4.2.9. Myorelaxants

Muscle relaxants are used to reduce skeletal muscle tone and, therefore, may be helpful in the management of TMD of muscular origin and chronic orofacial pain [164]. The most common myorelaxants include cyclobenzaprine, metaxalone, methocarbamol, and carisoprodol. Based on numerous studies, cyclobenzaprine is considered to be the drug of choice due to relieving the pain of muscular origin and improving sleep quality [165].

Caution should be exercised when using this type of medication due to its potential to induce significant sedation. These drugs are contraindicated in patients with hyperthyroidism, heart failure, after myocardial infarction, and heart rhythm disorders. The recommended dose is 10 mg at bedtime for 30 days, followed by a 2-week period to flush the drug out of the system and a medical follow-up. In the course of the therapy, the patient should always remain under medical supervision.

#### 4.2.10. Anticonvulsants

When discussing anticonvulsants, it is worth noting gabapentin, a GABA analogue. Gabapentin is thought to inhibit neurotransmitter release and reduce postsynaptic excitability [166].

The use of gabapentin reduces the pain of muscular origin, particularly from the temporal and masseter muscles. The drug is generally well tolerated and is associated with transient and mild side effects, including dizziness, drowsiness, dry mouth, weight gain, and impaired concentration [167].

#### 4.2.11. Benzodiazepines

Benzodiazepines facilitate transmission in the GABAergic system. They have been found to produce anxiolytic, sedative, hypnotic, anticonvulsant, and myorelaxant effects. Due to the risk of tolerance and dependence, as well as side effects including confusion, amnesia, and impaired motor coordination, these drugs are not recommended for the treatment of TMD [168].

## 5. Summary

Based on the literature review, the authors concluded that there is no single, ideal form of pain therapy for TMD. Treatment of TMD should be based on a thorough diagnostic process, including the DC/TMD examination protocol, psychological evaluation, and CBCT imaging. Following the diagnostic process, once a diagnosis is established, a treatment plan can be constructed to address the patient's complaints.

The treatment of temporomandibular dysfunctions requires a thorough diagnostic process, taking into account the etiology of the disorder. Having reviewed the relevant literature, the authors emphasize the need to combine multiple methods. For severe pain, pharmacotherapy may be used, while in other cases, it will be more appropriate to apply a combination of splint therapy and physiotherapy. While waiting for a custom-tailored occlusal splint, the patient can take advantage of behavioural and psychological methods, which should be continued after they have been fitted with the splint, as well as during physiotherapy treatments. Follow-up visits are an essential part of the TMD treatment process. The first follow-up visit should take place after one month of therapy and the next after three months. In the meantime, the patient should keep a diary describing their symptoms, pain levels, sleep quality, and wellbeing upon awakening and at bedtime. These observations, which should be reviewed at the follow-up visit, help build a full picture of the effects of the splint and other treatments, as well as inform the psychological assessment of the patient. An accurate diagnosis of TMD is the foundation of appropriate treatment. The most commonly described treatments include physiotherapy, occlusal splint therapy, and pharmacological treatment tailored to the type of TMD.

## Data Availability

No data were used to support this study.
